# COVID-19 pandemic and adrenals: deep insights and implications in patients with glucocorticoid disorders

**DOI:** 10.1007/s12020-023-03411-w

**Published:** 2023-06-20

**Authors:** Alessia Cozzolino, Valeria Hasenmajer, John Newell-Price, Andrea M. Isidori

**Affiliations:** 1 Department of Experimental Medicine, Sapienza University of Rome, IT, Rome, Italy; 2 Department of Oncology and Metabolism, The University of Sheffield, Sheffield, UK; 3 Centre for Rare Diseases (ENDO-ERN accredited), Policlinico Umberto I, Rome, Italy

**Keywords:** Covid-19, Adrenals, Glucocorticoids, Adrenal insufficiency, Cushing’s syndrome

## Abstract

**Purpose:**

Coronavirus disease-19 (COVID-19) has spread throughout the world. It was initially defined as a potentially severe syndrome affecting the respiratory tract, but it has since been shown to be a systemic disease with relevant extrapulmonary manifestations that increase mortality. The endocrine system has been found to be vulnerable to COVID-19 infection. The current review aims to evaluate the available data on the impact of COVID-19 infection and treatment, as well as COVID-19 vaccines, on adrenal gland function, particularly in patients with GC disorders.

**Methods:**

A thorough search of published peer-reviewed studies in PubMed was performed using proper keywords.

**Results:**

Adrenal viral tropism and severe acute respiratory syndrome coronavirus 2 (SARS-CoV-2) replication in the adrenal glands have been demonstrated, and adrenal insufficiency (AI) is a rare, but potentially severe complication in COVID-19 disease, whose recognition can be difficult if only for the empirical treatments administered in the early stages. Glucocorticoid (GC) treatment have had a pivotal role in preventing clinical deterioration in patients with COVID-19, but long-term GC use may increase COVID-19-related mortality and the development of iatrogenic AI. Patients with GC disorders, especially AI and Cushing’s syndrome, have been identified as being at high risk of COVID-19 infection and complications. Published evidence suggests that AI patient awareness and proper education may help adjust GC replacement therapy appropriately when necessary, thereby reducing COVID-19 severity. The COVID-19 pandemic has had an impact on AI management, particularly in terms of adherence to patients’ care plans and self-perceived challenges. On the other hand, published evidence suggests that the clinical course of COVID-19 may be affected by the severity of hypercortisolism in patients with CS. Therefore, to ameliorate the risk profile in these patients, cortisol levels should be adequately controlled, along with careful monitoring of metabolic and cardiovascular comorbidities. To date, the COVID-19 vaccine remains the only available tool to face SARS-CoV-2, and it should not be treated differently in patients with AI and CS.

**Conclusion:**

SARS-CoV-2 infection has been linked to adrenal damage and AI is a rare complication in COVID-19 disease, requiring prompt recognition. Educational efforts and patient awareness may reduce COVID-19 severity in patients with AI. Control of cortisol levels and monitoring of complications may improve the clinical course of COVID-19 in patients with CS.

## Introduction

The coronavirus, known as severe acute respiratory syndrome coronavirus 2 (SARS-CoV-2), was identified in January 2020. Coronavirus disease-19 (COVID-19), as it is officially known, has spread throughout the world. It was initially defined as a potentially severe syndrome affecting the respiratory tract, but it has since been shown to be a systemic disease with relevant extrapulmonary manifestations that increase mortality [1, 2]. The involvement of many extrapulmonary organs is linked to the ubiquitous expression of angiotensin-converting enzyme 2 (ACE2), the receptor responsible for SARS-CoV-2 entry at the cellular level [2, 3]. SARS-CoV-2 enters the cell by the binding of its homotrimeric spike glycoprotein to ACE2 [4, 5]. Spike protein is composed of two subunits: S1 and S2. In the presence of transmembrane serine protease receptor 2 (TMPRSS2), the S1 subunit dissociates, inducing a conformational change that increases S2 subunit stability and leads to membrane fusion [4, 6]. Viral entry is associated with widespread endothelial damage and an altered immune response, which contribute to the multiple organ involvement in COVID-19 [2, 7].

In humans, ACE2 mRNA and TMPRSS2 mRNA are expressed in several endocrine glands, including the adrenal cortex, making it a potential target for SARS-CoV-2 [4, 8].

The current review aims to evaluate the available data on the effects of COVID-19 infection and treatment, as well as COVID-19 vaccines, on adrenal gland function, particularly in patients with glucocorticoid (GC) disorders.

## COVID-19 and adrenal tropism

Adrenal glands are vulnerable to sepsis-induced organ damage, and their high vascularization and blood supply make them particularly susceptible to endothelial dysfunction and hemorrhage. Accordingly, adrenal insufficiency (AI) secondary to acute adrenal infarction [9, 10] and adrenal hemorrhage due to SARS-CoV-2 infection [11, 12] have been described in case reports and series.

Recently, Elhassan et al. performed a systematic literature review and, concomitantly, conducted a survey through the Society for Endocrinology clinical membership to identify patients with COVID‐19‐related adrenal hemorrhage using a standardized data collection tool. The literature search yielded 13 cases of COVID‐19 infection‐related adrenal hemorrhage/infarction (10 bilateral) and the UK survey captured 4 additional cases (2 bilateral) [13].

Furthermore, two autopsy series revealed varying frequencies of adrenal damage, ranging from 20 to 42.9% of the postmortem studies performed [14, 15].

A comprehensive histopathological examination of adrenal tissue sections from 40 autopsies of COVID-19-related deaths was conducted, and evidence of cellular damage and small vessel vasculitis in the periadrenal fat tissue and adrenal parenchyma was found [16]. Concurrently, increased perivascular lymphoplasmacellular infiltration and mild erythrocyte extravasation were found. These findings suggest that the adrenal gland is a prominent target for viral infection and that cellular damage is a potential trigger predisposing to adrenal dysfunction [16].

Recently, Paul and colleagues examined the morphologic and molecular features of 21 adrenal glands obtained during autopsies from patients with COVID-19 and demonstrated adrenal viral tropism and SARS-CoV-2 replication associated with local inflammation and severe adrenal damage [17]. Further histopathologic examination revealed widespread microthrombosis and severe adrenal injury. The viral identification in the adrenal gland and inflammatory cell accumulation accompanied by inflammatory cell death suggest that SARS-CoV-2 facilitates the induction of adrenalitis. Therefore, severe adrenal damage may result in primary AI with dysregulation of the hypothalamic-pituitary-adrenal (HPA) axis and, finally, hypocortisolemia [17].

In critically ill patients, such as those with COVID-19, the HPA axis may be unable to produce enough corticosteroids, resulting in critical illness-related corticosteroid insufficiency (CIRCI), also known as relative AI, with evidence of central hypocortisolism. CIRCI refers to GC levels that are insufficient for the severity of the critical illness, causing a magnified systemic inflammatory response [18, 19]. Indeed, AI is a potentially severe although rare systemic complication in COVID-19, as described in few case reports [9, 11, 12, 20–22] and some observational studies [10, 23], requiring a prompt recognition. The frequency of adrenal viral tropism, and the consequences of intra-adrenal vascular damage, can hardly be estimated as they were masked, especially in the early days of the pandemic, by the empirical use of many different drugs, including high doses of GCs.

Given the central role of the adrenal glands in immunoregulation and the significant adrenal injury observed in COVID-19, monitoring adrenal function and the potential development of AI may be necessary both during the acute phase of infection and during recovery. Nevertheless, whether the adrenal damage directly causes AI in patients with COVID-19 or its complications (such as long-term COVID) is still debated.

## Glucocorticoid treatment for COVID-19 and tertiary adrenal insufficiency

GC treatment has been strongly suggested to be effective in preventing clinical deterioration in patients with COVID-19. The RECOVERY study, a controlled, open-label trial comparing a variety of possible treatments in patients hospitalized with COVID-19, found that a single daily dose of oral or intravenous dexamethasone (6 mg) for up to 10 days resulted in lower 28-day mortality among patients requiring invasive mechanical ventilation [24]. Furthermore, the STOIC (Steroids in COVID-19) study, an open-label, parallel-group, Phase 2 randomized controlled trial of inhaled budesonide, investigated whether inhaled GC would effectively treat early COVID-19, compared to usual care, in adults within 7 days of the onset of mild COVID-19 symptoms: the study’s findings revealed that early administration of inhaled budesonide reduced the need for urgent medical care and shortened the time to recovery after early COVID-19 [25].

Furthermore, the PRINCIPLE study, a multicenter, open-label, multi-arm, randomized, controlled, adaptive platform trial conducted remotely from a central trial site and at primary care centers in the UK, demonstrated that inhaled budesonide improved time to recovery while possibly reducing hospital admissions or deaths (though not meeting the superiority threshold) in patients with COVID-19 who are at higher risk of complications [26].

All of the previous studies concluded that GCs are effective in improving the outcomes of patients with COVID-19. These data have been confirmed by a Cochrane review published in 2022 that evaluated whether inhaled corticosteroids are an effective treatment for patients with mild COVID-19. The authors concluded with moderate certainty that in patients with confirmed COVID-19 and mild symptoms who can use inhaler devices, inhaled corticosteroids probably reduce the combined endpoint of hospitalization or death and increase the resolution of all initial symptoms by Day 14. Low-certainty evidence suggests that corticosteroids have little to no effect on all-cause mortality up to Day 30 and may shorten the time to symptom resolution. In contrast, it was impossible to determine whether inhaled corticosteroids increased or decreased serious adverse events due to heterogeneity in the way they were reported across the studies. Furthermore, they found low-certainty evidence that inhaled corticosteroids may reduce infections [27]. However, because of the induction of immune deficiency, increased risk of secondary infections, and development of iatrogenic AI, prolonged GC treatment may increase COVID-19-related mortality [16, 28–30]. In particular, patients treated with dexamethasone for severe COVID-19 risk developing tertiary AI, even though low to moderate doses, as used in the RECOVERY trial, are less likely to cause this than higher or more prolonged does [16]. Whether fatigue after recovery from COVID-19 is due in part to AI is not clear.

There are few studies evaluating HPA axis function during COVID-19 infection, particularly in patients treated with GC, and the results are inconclusive. Table 1 summarizes the main findings of the studies evaluating adrenal function in patients admitted for suspected or confirmed COVID-19 disease.

**Table 1:** Details of studies evaluating adrenal function in patients admitted for suspected or confirmed COVID-19 disease

First author, year (Reference No)	Country	N° patients	GC therapy	Outcomes	Cortisol cut-off^a^(nmol/L)	Main findings
Alzahrani et al. 2020 [31]	Saudi Arabia	28	None	Morning plasma cortisol, ACTH, and DHEAS on different days of hospital admission	<300	Nine patients (32%) had abnormal cortisol levels with corresponding ACTH levels within or below the normal range of the assay. When the disease was more severe, the patients had lower cortisol and ACTH levels.
Das et al. 2021 [32]	India	84 (35 group I, moderate-to-severe disease; 49 group II, mild disease)	None	Morning serum cortisol, ACTH, and DHEAS, within 24–48 h of admission	<414	In group I, 38.5% of patients had hypocortisolism vs 6.8% in group II (*p* = 0.012). All hypocortisolic patients had low or inappropriately normal ACTH levels.
Ahmadi et al. 2022 [33]	Iran	154	None	Morning serum cortisol and ACTH on the first or second day of admission and hospitalization outcome	NA	Serum cortisol levels were lower in expired patients (11.3 μg/dl) than in discharged patients (16.7 μg/dl, *p* < 0.01), with no difference in plasma ACTH levels. At multivariate logistic regression analysis, serum cortisol level was a factor affecting patient death (*p* = 0.048).
Yavropoulou et al. 2022 [34]	Greece	52 COVID-19 33 controls	None	Diurnal, evening and nocturnal salivary cortisol, plasma ACTH	NA	Morning salivary cortisol levels did not differ between the two groups, but patients exhibited higher median levels of evening and nocturnal salivary cortisol compared to controls (*p* < 0.001).
Clarke et al. 2021 [35]	UK	70	22 patients received dexamethasone during the acute phase of disease; none received GC following recovery	Short Synacthen test (250 μg IV bolus) Persistence of COVID-19 symptoms, such as fatigue	peak <450 after Synacthen	Adrenal function ≥3 months after presentation with COVID-19 was preserved in all patients, while a significant proportion of them (62.9%) experienced persistent fatigue.
Tan et al. 2020 [36]	UK	535 (403 COVID-19; 132 No COVID-19)	None	Cortisol levels within 48 h of admission	NA	Median cortisol concentration in COVID-19 group was 619 nmol/L vs 519 nmol/L in No COVID-19 group (*p* < 0·0001). In COVID-19 group, increased cortisol was predictive of mortality. Multivariable analysis showed that a doubling of cortisol level was associated with a significant 42% increase in the hazard of mortality (*p* = 0.014).
Guven et al. 2021 [37]	Brazil	285 (141 COVID-19; 144 No COVID-19)	None	Morning serum cortisol levels	NA	Median cortisol level was 21.84 μg/dl in COVID-19 group and 16.47 μg/dl in No COVID-19 group (*p* < 0.001). In COVID-19 group, cortisol level results as a significant factor influencing mortality on both univariate (*p* = 0.000) and multivariate (*p* = 0.001) logistic regression.

*GC* glucocorticoids, *ACTH* adrenocorticotropic hormone, *DHEAS* dehydroepiandrosterone sulfate, *NA* not available

^a^For abnormal adrenal function

Alzahrani and colleagues investigated the adrenocortical response to acute COVID-19 by measuring morning serum cortisol, plasma adrenocorticotropic hormone (ACTH), and serum dehydroepiandrosterone sulfate (DHEAS) levels in 28 consecutive patients on Days 1 and 2 of hospitalization, and in a subset of them the tests were repeated twice and thrice on different days. None of them received GC. Considering abnormal for an acute setting a cutoff cortisol of <300 nmol/L, the authors found that a significant proportion of the patients had cortisol levels below this limit and the corresponding ACTH levels were within or below their laboratory reference range, consistent with central AI. They also found that patients with more severe disease, had lower cortisol and ACTH levels [31].

Das and colleagues examined the HPA axis in 84 consecutive patients with COVID-19 to identify whether the degree of HPA axis dysfunction was related to disease severity. None of them received GC. The authors found that patients with moderate-to-severe disease had a higher prevalence of hypocortisolism (38.5% vs 6.8%, *p* = 0.012), and a trend toward lower ACTH and DHEAS levels than patients with mild disease [32].

These findings were partially confirmed by Ahmadi and colleagues on 154 hospitalized patients with COVID-19, who found that cortisol levels were substantially lower in non-survivors than in survivors, while ACTH levels were unaffected. According to a logistic regression model, circulating cortisol was one of the most important factors predicting mortality among patients not receiving GC [33].

A recent study by Yavropoulou et al. reported the findings on 52 patients with COVID-19, evaluated before dexamethasone treatment when this was needed, and compared with 33 healthy age- and sex-matched controls. The authors found that the circadian rhythm of free bioavailable cortisol was blunted in patients with mild or moderate COVID-19, with higher evening and nocturnal cortisol levels, while salivary morning cortisol levels remained unaltered [34].

Clarke et al. assessed adrenal function in a prospective, observational study of 70 patients at least 3 months after their COVID-19 diagnosis. Patients who were prescribed GC treatment after COVID-19 recovery were excluded from the study. Dexamethasone was given to 31.4% of the 70 COVID-19 survivors, as part of their acute treatment for COVID-19. Participants underwent a short Synacthen test, and their adrenal function was found to be normal 3 months or more after COVID-19 diagnosis. However, a significant proportion of patients experienced persistent fatigue that was unrelated to alterations in adrenal function [35]. These findings reassured about the risk of medium-term adrenal dysfunction following dexamethasone administration according to the RECOVERY protocol (6 mg once daily for at least 10 days), which was used in a large number of patients with COVID-19.

A previous study from the same group demonstrated that in the acute setting, patients with COVID-19 had significantly higher cortisol levels taken within 48 h of admission than patients without and that increased cortisol was predictive of mortality. Furthermore, an appropriate stress response was found during acute COVID-19 without any evidence of acute AI [36].

Similarly, Guven et al. found that patients with COVID-19 had higher cortisol levels than patients without and that cortisol levels were associated with increased risk of death in patients with COVID-19 in the intensive care unit [37].

These conflicting results across studies could be explained by the different populations included as well as the different methods used to assess adrenal function; moreover, the quality of evidence is low for the great majority of them. A number of biases can hamper the meaning of these studies: validity of assays, concomitant medications, comorbidities, time of sampling, and more. However, as the often unpredictable evolution of SARS-Cov2 infection has taught, with some patients improving and other rapidly worsening, the possibility that in some patients the HPA responded appropriately, and in others failed to counteract viral spread and immune overeaction, cannot be ruled out.

Currently, it is impossible to estimate the true AI incidence in patients with COVID-19, particularly those receiving long-term GC treatment, because of the scarcity of studies evaluating the HPA axis function.

To date, only one study [35] has assessed adrenal function in patients with COVID-19 using the standard 250 μg Synacthen test, which remains the most widely used dynamic function test for assessing the integrity of the HPA axis and is the gold-standard test for diagnosing primary AI [38, 39], and found it preserved.

Nevertheless, in patients with COVID-19 who need to continue GC therapy for several weeks, regardless of infection status, assessment of adrenal reserve is needed.

## COVID-19 in patients with adrenal insufficiency

Physiological GC concentrations play a pivotal role in priming the immune system during the early stages of an infection. Since patients with known AI are at high risk of infection due to their depleted innate immunity [40], it was speculated at the beginning of the COVID-19 pandemic that impairment of immune function in such patients may make them especially susceptible to adverse outcomes [28, 40]. Therefore, several experts from various endocrinological societies and countries promptly published recommendations on the management of steroid replacement therapy in patients with AI during the COVID-19 pandemic [41, 42].

The European guidelines for AI management during the COVID-19 pandemic highlight the importance of patient education (sick day rules, strict social distancing rules), equipment (sufficient GC supplies, steroid emergency self-injection kit), and empowerment (steroid emergency card, COVID-19 guidelines) in preventing adrenal crises. In the case of acute COVID-19 infection with continuous high fever, they recommend an oral stress dose covered with 20 mg hydrocortisone every 6 h (sick day rule 1). Patients on modified-release hydrocortisone should be switched to immediate-release hydrocortisone. Patients taking oral prednisolone should divide their dosage into two equal doses of at least 10 mg each. In the case of clinical deterioration, they recommend immediate self-injection of 100 mg hydrocortisone intramuscularly, followed by continuous intravenous infusion of 200 mg hydrocortisone every 24 h, or until this can be established, and administration of 50 mg hydrocortisone every 6 h (sick day rule 2) [43]. This regimen can reduce the harmful effects of hydrocortisone peaks and troughs on the immune system [44, 45] as well as the length of stay in an intensive care unit [44, 46]. Concurrently, hydration and electrolyte balance should be maintained to prevent the severe hypotension that can occur as the disease progresses [44].

In the case of mildly symptomatic COVID-19, given that hydrocortisone clearance decreases with stress, it has been suggested that it is safe to replace the missing stress-induced cortisol rise with additional doses, at least doubling the original regimen [44, 45].

In addition, severe COVID-19 has been linked to disseminated thromboembolic diseases. Given the coagulation abnormalities associated with GC use, it seems reasonable to introduce low molecular weight heparin as soon as possible [44, 47].

Unexpectedly, 3 years after the pandemic began, published evidence suggests that, if educational efforts are made properly to prevent acute events, patients with AI may not be particularly susceptible to COVID-19 [48, 49]. Table 2 summarizes the main findings of the studies evaluating COVID-19 prevalence and symptoms in patients with AI.

**Table 2:** Details of studies evaluating COVID-19 prevalence and symptoms in patients with adrenal insufficiency

First author, year (Reference No)	Country	Study design	N° patients	COVID-19 prevalence^a^	Outcomes	Main findings
Martino et al. 2020 [48]	Italy	Monocentric cross-sectional study	121 (40 PAI; 81 SAI)	0.8%	Three questionnaires: 1. CORTI-COVID 2. AddiQoL-30 3. SF-36 evaluating pandemic-related psychophysical stress, lockdown-induced emotional burden, and health-related QoL	No AC reported. Mean CORTI-COVID was similar between groups, mainly depending on “personal health” in PAI and “economy” in SAI. Working restrictions increased occupational concern. CORTI-COVID correlated inversely with QoL. AddiQoL-30 and SF-36 correlated strongly. Comorbidities worsened patients’ QoL.
Carosi et al. 2021 [50]	Italy	Retrospective case-control study	279 (60 PAI; 219 SAI) 112 controls	0.7% AI 0% controls	Standardized questionnaire by phone evaluating COVID-19 suggestive symptoms and consequences	The prevalence of symptomatic patients was similar between the 2 groups. Highly suggestive COVID-19 symptoms (at least 2 including fever and/or cough) also occurred equally in AI and controls. No patient required hospitalization and no AC reported.
Graf et al. 2021 [51]	UK	Cross-sectional study	159 SAI	1.2%	A telephone survey evaluating direct and indirect impact of the COVID-19 pandemic	64.8%^b^ of patients experienced a delay or change in the planned care for their pituitary disease, with 24.3%^b^ of patients perceiving an impact to their care.
Li et al. 2021 [52]	USA	Prospective longitudinal survey study	342 (157 PAI; 109 SAI; 76 GIAI)	1.2%	Patient-centered questionnaire (Depression Anxiety Stress Scales-21, SF-36, and AI self-management) evaluating the impact of the pandemic on self-reported outcomes	Patients reported a higher financial impact from AI and difficulty accessing medical care during the pandemic. A third of patients reported difficulty managing AI during the pandemic. Younger patients, women, poor healthcare access, lack of good insurance support, and those with a higher financial impact reported greater difficulties managing AI.
Nowotny et al. 2023 [53]	Europe	European multicentre questionnaire	57 (45 PAI; 7 SAI; 5 GIAI)	NA	Questionnaire evaluating the outcome of COVID-19 infection in patients with adrenal disorders	An increase by a median of 2 times the daily replacement dose was reported in 74% patients and the use of emergency GC injection in 3.5%; hospitalization was required in 8.8% of patients due to AC and in 7.0% due to severity of COVID-19 infection.

*PAI* primary adrenal insufficiency, *SAI* secondary adrenal insufficiency, *AC* adrenal crisis, *GIAI* glucocorticoid induced adrenal insufficiency, *GC* glucocorticoids, *NA* not applicable

^a^Prevalence refers to COVID-19 diagnosis obtained by a nasopharyngeal swab positive test, calculated on the total number of patients

^b^Percentages refer to the whole population of 412 patients with pituitary disease

To assess the prevalence and clinical presentation of COVID-19, the prevalence of adrenal crisis, and its association with COVID-19 or pandemic-related psychophysical stress, lockdown-induced emotional burden, and health-related quality of life (QoL) in a cohort of 121 patients with AI, Martino and colleagues used the remote completion of three questionnaires: (1) the purpose-built CORTI-COVID, which assesses the medical history and concerns for COVID-19-related global health and AI-specific personal health, occupational, economic, and social consequences; (2) the AddiQoL-30; and (3) the Short-Form-36 Health Survey. They concluded that the novel CORTI-COVID questionnaire is a reliable tool for assessing pandemic-related emotional burdens in AI. Furthermore, they found that even under unusual stress, patients’ education can help them maintaining a high QoL [48].

Carosi et al. conducted a retrospective case-control study to assess the incidence of COVID-19 symptoms and complications in patients with AI using a phone-administered standardized questionnaire. The study enrolled 279 patients and 112 controls and found that the prevalence of symptomatic patients—those who complained of at least one viral infection symptom—was comparable between the two groups. Similarly, at least two highly suggestive COVID-19 symptoms, including fever and/or cough, occurred equally in patients and controls. The study’s findings confirmed that patients with AI who have been adequately treated and trained seem to have the same incidence of COVID-19-suggestive symptoms and disease severity as controls [50].

Graf et al. conducted a cross-sectional study on 412 patients with pituitary disease (159/412 with secondary AI) via a telephone survey. Although only a few patients had confirmed or suspected SARS-CoV-2 infection, the pandemic had affected more than half of them, causing a delay or change in their planned care. The vast majority of the 159 patients with AI were adequately informed about sick day management, which may explain why the effect of COVID-19 infection on these patients was partially mitigated [51].

Li et al. conducted a prospective longitudinal survey study at two tertiary centers using a patient-centered questionnaire to assess the impact of the pandemic on self-reported outcomes in patients with AI. The daily GC dose and the number of adrenal crises did not change during the pandemic from pre-pandemic status. However, one-third of patients reported difficulties with AI management during the pandemic, particularly among younger patients, women, and those with limited access to healthcare facilities [52].

Recently, Nowotny et al. reported the results of a European multicentre questionnaire aiming at evaluating the outcome of COVID-19 infection in patients with adrenal disorders and including 57 patients with AI (45 primary AI; 7 secondary AI; 5 tertiary AI). In this cohort, an increase by a median of 2 times the daily replacement dose was reported in 74% of patients and the use of emergency GC injection was reported in 3.5% of patients. Hospitalization was required in 8.8% of patients due to adrenal crises and in 7.0% due to severity of COVID-19 infection. Overall, the results of the study suggest good clinical outcomes in case of duly dose adjustments and emphasize the importance of patient education on sick day rules [53].

In summary, published evidence suggests that patient awareness and proper education may help adjust GC replacement therapy appropriately when necessary, thereby reducing COVID-19 severity. Furthermore, lower SARS-CoV-2 infection rates in patients with AI may be explained by increased vigilance and better adherence to state- or nation-wide social distancing and stay-at-home policies. Regarding the COVID-19 prevalence in these patients, it should be noted that all of the studies are limited by a lack of specific SARS-CoV-2 diagnostic data, as nasopharyngeal swabs were only performed in specific situations, namely hospital admissions, at the time of the aforementioned studies.

In addition, previous studies suggest that the pandemic has had an impact on AI management, particularly in terms of adherence to patients’ care plans [51], as well as self-perceived challenges, most likely due to pandemic-related increases in childcare responsibilities, lockdown measures, furloughs, and economic burden, as well as possible difficulties with telemedicine access [52].

## COVID-19 in patients with endogenous Cushing’s syndrome

COVID-19 has been linked to a high rate of infection and mortality, particularly in patients with comorbidities such as obesity, hypertension, diabetes, and immunodeficiency syndromes [54].

Cushing’s syndrome (CS), a rare endocrine disease characterized by excessive GC secretion, which causes cardiometabolic and immune impairment, shares some of these comorbidities that lead to increased COVID-19 morbidity and mortality. Moreover, the increased susceptibility to cardiovascular and thromboembolic disorders as well as severe infections are the main causes of death in patients with CS [55].

GC excess affects all immune cells, resulting in overall immune suppression with cell death, impaired immune regulation, and a defective immune response. CS-induced lymphopenia, mainly of the CD4+ subset with an altered CD4/CD8 ratio, makes patients more susceptible to viral infections ([55, 56]. Thus, patients with CS may be at high risk of developing a SARS-CoV-2 infection with severe clinical outcomes [1, 57]. As a result, several recommendations for the clinical management of patients with endogenous CS were published in response to the COVID-19 pandemic [58, 59].

First of all, the authors recommend treating comorbidities as a standard of care and avoiding the use of ACE inhibitors or angiotensin II receptor type 1 blockers for hypertension treatment due to the potential impact on susceptibility to the SARS-CoV-19 infection. Furthermore, prophylaxis for Pneumocystis jirovecii with trimethoprim/sulfamethoxazole is highly recommended in patients with severe CS. In patients with cough, fever, and respiratory distress, a differential diagnosis between the SARS-CoV-19 infection and other respiratory infections is required to ensure appropriate treatment. Because of the high thromboembolic risk in these patients due to both CS and COVID-19, low molecular weight heparin is recommended until definitive treatment is achieved.

Steroidogenesis inhibitors are recommended as the mainstay treatment for hypercortisolism because they are effective for all types of CS. A GC receptor antagonist may also be considered, but its use is limited due to difficult titration and limited availability. In contrast, dopamine agonists and somatostatin analogs are not recommended as monotherapy for patients requiring immediate biochemical control.

In addition, it is suggested that when using steroidogenesis inhibitors, patients should be considered for a “block and replace” approach to reduce the risk of AI. They should also have access to stress doses of GC tablets and an emergency injection kit, and they should be aware of the “sick day rules” and the importance of taking extra GC when necessary. Regular phone or video consultations are recommended to assess treatment response and hypoadrenalism symptoms [58, 59].

The published data on endogenous CS and COVID-19 are limited. Table 3 summarizes the studies and case reports that evaluated the prevalence and progression of COVID-19 in patients with endogenous CS.

**Table 3:** Details of studies evaluating COVID-19 prevalence and symptoms in patients with endogenous Cushing’s syndrome

First author, year (Reference No)	Country	Study design	N° patients	COVID-19 prevalence^a^	Outcomes	Main findings
Belaya et al. 2020 [60]	Russia	Observational study	22	9.1%	Clinical course and laboratory characteristics of patients with CS and COVID-19 infection	Severe CS affected by COVID-19 is more likely to require emergency care despite a lack of clinical presentations and low inflammation biomarkers.
Serban et al. 2021 [61]	Italy	Monocentric cross-sectional study	61 CD 61 controls	3.2% CS 0% controls	Telephone survey collecting information related to influenza vaccination, risky behaviors for COVID-19, suggestive clinical features and COVID-19 testing	Almost 38% of CS and 47% of controls had at least one clinical feature between January and mid-April. A severe clinical presentation was observed, especially in patients with active CS.
Yuno et al. 2021 [62]	Japan	Case report	1	NA	Clinical course of COVID-19 pneumonia in a patient with active CD	Medical treatment of hypercortisolism with metyrapone by the ‘block and replace’ regimen using hydrocortisone. The COVID-19 pneumonia improved with multi-modal treatment. After a negative SARS-CoV-2 test result trans-sphenoidal surgery was performed.
Nowotny et al. 2023 [53]	Europe	European multicentre questionnaire	7 (3 CD; 4 ACS)	NA	Questionnaire evaluating the outcome of COVID-19 infection in patients with adrenal disorders	43% of patients were hospitalized due to the severity of COVID-19 infection; 1/7 patients (14.3%) had a fatal outcome, a 60-year-old female with CD on treatment with ketoconazole with multiple comorbidities, passed away after 30 days of ICU treatment.
Ragonese et al. 2023 [63]	Italy	Observational study	60 CD	11.7%	Epidemiology, course, and outcomes of COVID-19 disease	Mean age of infected patients was significantly lower than the unaffected ones, but sex, BMI, or diabetes mellitus were not risk factors for infection/worse outcomes. None of patients died.

*CS* Cushing’s syndrome, *CD* Cushing’s disease, *NA* not applicable, *ACS* adrenal Cushing’s syndrome, *ICU* Intensive care unit

^a^Prevalence refers to COVID-19 diagnosis obtained by a nasopharyngeal swab positive test, calculated on the total number of patients

Belaya et al. conducted an observational study on 22 patients with CS, evaluating the clinical course and laboratory characteristics of those with COVID-19. They detected COVID-19 in 9.1% of their cohort and found that severe CS affected by COVID-19 were more likely to require emergency care despite a lack of clinical manifestations and low inflammation biomarkers [60].

Serban et al. conducted a monocentric cross-sectional study on 122 patients (61 patients with Cushing’s disease (CD) and 61 controls) using a telephone survey to collect data on influenza vaccination, risky COVID-19 behaviors, suggestive clinical features, and COVID-19 testing. They found two cases (3.2%) of virologically confirmed COVID-19 in patients with CD but none in the control group. Furthermore, nearly 38% of CDs and 47% of controls had at least one clinical feature suggestive of COVID-19 between January and mid-April. A severe clinical presentation was observed, particularly, in patients with active CD [61].

Yuno et al. described the course of COVID-19 pneumonia in a 27-year-old female patient with active CD. The “block and replace” regimen with metyrapone and hydrocortisone was used to treat hypercortisolism, and COVID-19 pneumonia improved on multi-modal treatment. After a negative SARS-CoV-2 test result, trans-sphenoidal surgery was successfully completed [62].

Recenlty, Nowotny et al. reported the results of the European multicentre questionnaire including also 7 patients with CS (3 CD; 4 adrenal CS). In this cohort, 43% of patients were hospitalized due to the severity of COVID-19 infection and among them one had a fatal outcome, a 60-year-old female with CD on treatment with ketoconazole, with multiple comorbidities [53].

Ragonese et al. investigated epidemiology, course, and outcomes of COVID-19 infection in 60 patients with CD, being the majority of them (43/60, 71.7%) with controlled disease, and found a COVID-19 prevalence comparable with general population (11.6% vs 14.7%). Moreover, they found SARS-CoV-2-positive patients having a lower average age than unaffected patients, but sex, BMI, or diabetes mellitus did not seem to influence disease susceptibility or worsen the outcome in their cohort [63].

Although the evidence is limited, published evidence suggests that the clinical course of COVID-19 may be affected by the severity of hypercortisolism in patients with CS, and patients with both florid CS and COVID-19 are more likely to require emergency care. Therefore, to ameliorate the risk profile of patients with CS, hypercortisolism should be adequately controlled, along with careful monitoring of metabolic and cardiovascular comorbidities.

## COVID-19 vaccination in patients with glucocorticoid disorders

Vaccine availability has been a crucial step in combating the COVID-19 pandemic. Two clinical trials comparing the efficacy and safety of the mRNA-1273 SARS-CoV-2 and mRNA-BNT162b2 COVID-19 vaccines in patients with stable diabetes and obesity to healthy subjects yielded comparable results [64, 65]. Consequently, the European Society of Endocrinology (ESE) issued a statement recommending that COVID-19 vaccination not be treated differently in patients with stable endocrine diseases, such as Addison’s disease or pituitary adenomas, than in the general population, and that patients with AI be informed to adjust GC replacement therapy appropriately in case of side effects [2]. The most common side effects of mRNA vaccines are pain for several days at the injection site, fatigue, headache, myalgias, arthralgias, chills, and fever [64, 65], and all of these signs and symptoms can early indicate an increased need for GC replacement therapy in patients with AI [66].

The Pituitary Society surveyed its membership to learn about planned approaches to GC management in patients with AI who would receive a COVID-19 vaccine, aiming to disclose suggested best practices to the endocrinological community based on responses from experienced clinicians treating pituitary diseases. A total of 103 members responded to the survey. The majority (64%) did not intend to recommend an automatic GC dose increase with vaccination but only in cases of fever, and half suggested increasing the GC dose also in cases of arthralgias and myalgias [66].

Pilli and colleagues recently conducted a prospective study to assess the tolerability and need for GC dose adjustment in a cohort of patients with AI receiving COVID-19 mRNA vaccines. A questionnaire about the occurrence, severity, and duration of side effects, as well as the need for replacement therapy dose adjustment 1 week after the first and second doses of COVID-19 mRNA vaccines, was given to 88 patients with AI (28 with primary and 60 with secondary AI). Side effects of mild to moderate severity occurred in about 70% of patients following both vaccine doses, with pain at the injection site, fatigue, fever, and flu-like symptoms being the most common adverse events. No correlation was found between gender, AI type, and the mRNA vaccine, but the prevalence of adverse events increased after the second dose. Up to 8% of patients required doubling their oral GC dose, with no need for parenteral administration. Thus, the authors concluded that COVID-19 mRNA vaccines were well tolerated in these patients, with tolerable side effects when compared to the general population, with no need to increase replacement therapy dosage prior to vaccination [67].

A case series of five adrenal crises occurring in patients with AI (3 with primary and 2 with secondary AI) within the first 24 h after the first dose of the AstraZeneca ChAdOx1 SARS‐CoV‐2 vaccine was published, and the authors concluded that the temporal association strongly suggests that the COVID‐19 vaccine was a plausible precipitant of adrenal crises [68]. In addition, a case report described the occurrence of an adrenal crisis in a patient with AI even after COVID-19 mRNA vaccination, suggesting that different types of COVID-19 vaccines may precipitate adrenal crises in these cohorts [69].

Previous cases reported in the literature underline the importance of increasing the maintenance dose of GC in patients with AI who exhibit any symptoms after COVID-19 vaccination.

On the other hand, vaccine-induced immune thrombotic thrombocytopenia (VITT) with venous thrombosis is a rare complication of SARS-CoV-2 vaccination with ChAdOx1 (AstraZeneca) and AD26.COV2.S (Johnson & Johnson), and it is associated with high mortality [70, 71]. The main hypothesis for the pathophysiology of immune thrombotic thrombocytopenia induced by an adenoviral vector-based COVID-19 vaccine involves the reaction between cationic platelet factor 4 (PF4) and anionic-free DNA in the recombinant adenovirus vaccine [72]. PF4 antibodies cause VITT by activating platelets and coagulation, leading to thrombosis [73].

When thromboembolic events affect the adrenal veins, adrenal hemorrhage and AI occur. To the best of our knowledge, sixteen cases of adrenal bleeding followed by AI after COVID-19 vaccination have been reported to date: thirteen after AstraZeneca [13, 74–82], two after Pfizer [13] and one after Johnson & Johnson immunization [83]. Furthermore, one day after receiving the second dose of the BNT162b2 SARS-CoV-2 vaccine, an isolated ACTH deficiency has been described, with an adrenal crisis and magnetic resonance images revealing an atrophic pituitary gland [84].

Finally, while AI does not preclude COVID-19 vaccination, vaccination side effects must be carefully assessed to promptly adjust GC replacement therapy and avoid adrenal crises in these patients.

## Conclusions

The endocrine system has been found to be vulnerable to COVID-19 infection. Adrenal viral tropism and SARS-CoV-2 replication, in particular, have been linked to local inflammation and severe adrenal damage. Indeed, AI is a potentially severe, although rare, systemic complication in COVID-19, requiring prompt recognition.

Although long-term GC treatment may increase COVID-19 mortality and iatrogenic AI development, it has been strongly suggested to be effective in preventing clinical deterioration in patients with COVID-19. Currently, it is impossible to estimate the true incidence of AI in patients with COVID-19, particularly those receiving long-term GC treatment, because studies evaluating the HPA axis function are limited. Nevertheless, assessing the risk of developing AI is critical to avoid poor monitoring in outpatient settings or inadequate tapering.

Patients with AI have been considered to be at a high risk of SARS-CoV-2 infection and complications due to their depleted innate immunity. Published evidence suggests that patient awareness and proper education may help adjust GC replacement therapy appropriately when necessary, thereby reducing COVID-19 severity. Furthermore, increased vigilance and adherence to social distancing and stay-at-home policies may have reduced COVID-19 rates in patients with AI. The COVID-19 pandemic has had an impact on AI management, particularly in terms of adherence to patients’ care plans and self-perceived challenges.

On the other hand, patients with hypercortisolism have been considered to be at high risk of developing COVID-19 infection with severe clinical outcomes. Published evidence suggests that the clinical course of COVID-19 may be affected by the severity of hypercortisolism. Therefore, to ameliorate the risk profile in patients with CS, cortisol levels should be adequately controlled, along with careful monitoring of metabolic and cardiovascular comorbidities.

To date, the COVID-19 vaccine remains the only available tool to face SARS-CoV-2, and it should not be treated differently in patients with endocrine diseases, particularly AI and CS. Although AI does not preclude COVID-19 vaccination, vaccine side effects must be carefully assessed to promptly adjust GC replacement therapy and avoid adrenal crises in these patients.
